# Spatial-temporal detection of risk factors for bacillary dysentery in Beijing, Tianjin and Hebei, China

**DOI:** 10.1186/s12889-017-4762-1

**Published:** 2017-09-25

**Authors:** Chengdong Xu, Yuanyuan Li, Jinfeng Wang, Gexin Xiao

**Affiliations:** 1 State Key Laboratory of Resources and Environmental Information System, Institute of Geographic Sciences and Natural Resources Research, Chinese Academy of Sciences, Beijing, China; 2 Chang’an University, Xi’an, China; 3 China National Center for Food Safety Risk Assessment, Beijing, China

**Keywords:** Bacillary dysentery, Meteorological factors, Socio-economic factors, Spatial-temporal, GeoDetector model

## Abstract

**Background:**

Bacillary dysentery is the third leading notifiable disease and remains a major public health concern in China. The Beijing–Tianjin–Hebei urban region is the biggest urban agglomeration in northern China, and it is one of the areas in the country that is most heavily infected with bacillary dysentery. The objective of the study was to analyze the spatial-temporal pattern and to determine any contributory risk factors on the bacillary dysentery.

**Methods:**

Bacillary dysentery case data from 1 January 2012 to 31 December 2012 in Beijing–Tianjin– Hebei were employed. GeoDetector method was used to determine the impact of potential risk factors, and to identify regions and seasons at high risk of the disease.

**Results:**

There were 36,472 cases of bacillary dysentery in 2012 in the study region. The incidence of bacillary dysentery varies widely amongst different age groups; the higher incidence of bacillary dysentery mainly occurs in the population under the age of five. Bacillary dysentery presents apparent seasonal variance, with the highest incidence occurring from June to September. In terms of the potential meteorological risk factors, mean temperature, relative humidity, precipitation, mean wind speed and sunshine hours explain the time variant of bacillary dysentery at 83%, 31%, 25%, 17% and 13%, respectively. The interactive effect between temperature and humidity has an explanatory power of 87%, indicating that a hot and humid environment is more likely to lead to the occurrence of bacillary dysentery. Socio-economic factors affect the spatial distribution of bacillary dysentery. The top four factors are age group, per capita GDP, population density and rural population proportion, and their determinant powers are 61%, 27%, 25% and 21%, respectively. The interactive effect between age group and the other factors accounts for more than 60% of bacillary dysentery transmission.

**Conclusions:**

Bacillary dysentery poses a higher risk in the population of children. It is affected by meteorological and socio-economic factors, and it is necessary to pay more attention to the meteorological period and situation, particularly in period with high temperature and humidity, as well as places in urban areas with high population density, and a low proportion of rural population.

## Background

Bacillary dysentery is an intestinal infectious disease caused by different types of shigella. It is mainly spread by carriers such as faeces, hand pollution, food, water, flies, or cockroaches and other indirect means of transmission, and ultimately from the mouth into the digestive tract, which is susceptible to infection. Clinical manifestations are chills, fever, abdominal pain, diarrhoea, and so on [1]. Bacillary dysentery remains a public health concern, in developing countries [2–4]. In China, the disease burden of bacillary dysentery is still severe [5]. According to the China National Disease Surveillance System, there were 205,000 reported cases of bacillary dysentery in 2012, amongst which, the incidence of bacillary dysentery in Beijing and Tianjin was 65.27 per 100,000 population and 63.76 per 100,000 population, respectively, ranking in the top two in China [6].

The epidemic features of bacillary dysentery present apparently temporal variation; it mainly occurs in the summer and autumn in the North of China [7, 8]. There are studies showing that the temporal variant of bacillary dysentery is associated with meteorological factors; for example, it suits bacillary dysentery to replicate and survive in a higher-temperature environment [9]. Bacillary dysentery incidence increases by 14.8% with a 1 °C increase in mean temperature in Changsha city [10]. Meanwhile, precipitation is also found to be associated with transmission of bacillary dysentery; the excess risk of bacillary dysentery incidence increases by 0.22% with a 1 mm increase in precipitation in Beijing [11].

Besides temporal variation being a factor for bacillary dysentery, it also presents apparent spatial heterogeneity. This is mainly caused by the spatial variant of socio-economic factors [12, 13], because overcrowded environments with poor sanitation are conducive for the transmission of bacillary dysentery. In China, socio-economic development is regionally unbalanced. In the study region, Beijing and Tianjin are developed areas, but surrounded by some impoverished areas. Beijing-Tianjin-Hebei is located in a monsoon climate zone with high climatic variations; for example, higher temperature and precipitation are mainly present in summer and autumn. Meanwhile, there are significant socio-economic differences in geographic space. In the study, a novel spatial-temporal method, the GeoDetector, was used to identify potential socio-economic and meteorological risk factors associated with bacillary dysentery in time and space dimensions, this will be expanded in the methods section.

## Methods

### Materials

#### Bacillary dysentery

Daily bacillary dysentery cases from 1 January 2012 to 31 December 2012 in Beijing–Tianjin– Hebei were employed. Bacillary dysentery data were obtained from the daily real-time reported system from the Chinese Centre for Disease Control and Prevention. Disease incidence was calculated by determining the ratio between the number of cases with bacillary dysentery and the population size of a given county or age group.

**Fig. 1:**
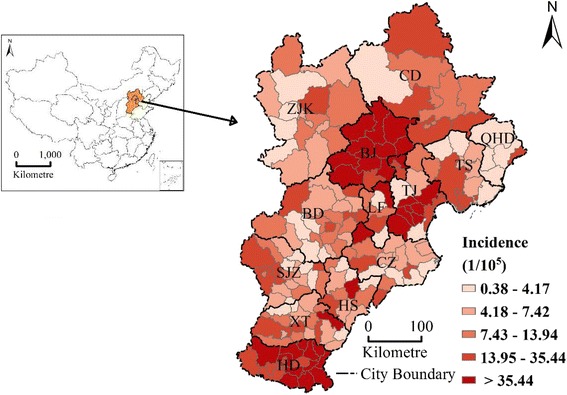
Geographic location of the Beijing–Tianjin–Hebei area in China and disease rate of bacillary dysentery in the study area

Figure 1 shows that the spatial distribution of bacillary dysentery incidence presents apparent spatial heterogeneity, and the area with the highest incidences are mainly located in Beijing, Tianjin and the southern regions of Hebei province.

### Potential risk factors

Both environmental and economic factors affect the incidence of bacillary dysentery; potential risk factors, which include socio-economic variables and meteorological climate, were considered in the study. These socio-economic data, including the variable of medical and technical personnel, in each county were obtained from the 2012 Statistical Yearbook [14–16]. These daily meteorological data were obtained from the China Meteorological Data Sharing Service System in 27 meteorological stations distributed in the Beijing–Tianjin–Hebei region from 1 January 2012 to 31 December 2012.

**Fig. 2:**
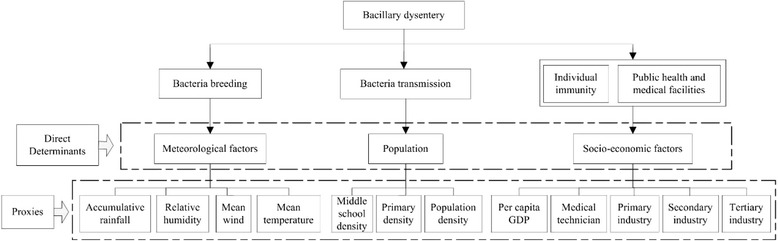
Determinants of bacillary dysentery and their proxies

Bacillary dysentery spread is affected by multiple aspects, including the environment for bacteria breeding and transmission, individual hygiene conditions and public health medical facilities (Fig. 2). The variables collected in the study accord with the relationship between bacillary dysentery and potential factors.

One of the most important factors that could affect the incidence of bacillary dysentery is the pathogenic bacterial environment, including the bacterial breeding environment and bacterial transmission environment. There have been many studies focused on the impact of potential risk factors [13, 17–20]. For the bacterial breeding environment, mean temperature, precipitation, relative humidity and mean wind speed were selected as proxy variables; Another important factor is that affecting the transmission of bacillary dysentery, for which, population density and industry structure were chosen as proxy variables. The industry structure data includes: primary, secondary, and tertiary industry. The primary sector is dominated by the production of raw materials, for example, farming and fishing. The secondary sector undertakes industrial manufacturing (making products using the raw materials extracted by the primary sector businesses). The tertiary sector is involved in selling the products made by secondary sector businesses and also sell services. Goods are not manufactured by tertiary sector businesses. Individual health and medical facilities are reflected by proxy variables of per capita global domestic product (GDP) and medical and technical personnel per thousand persons.

**Fig. 3:**
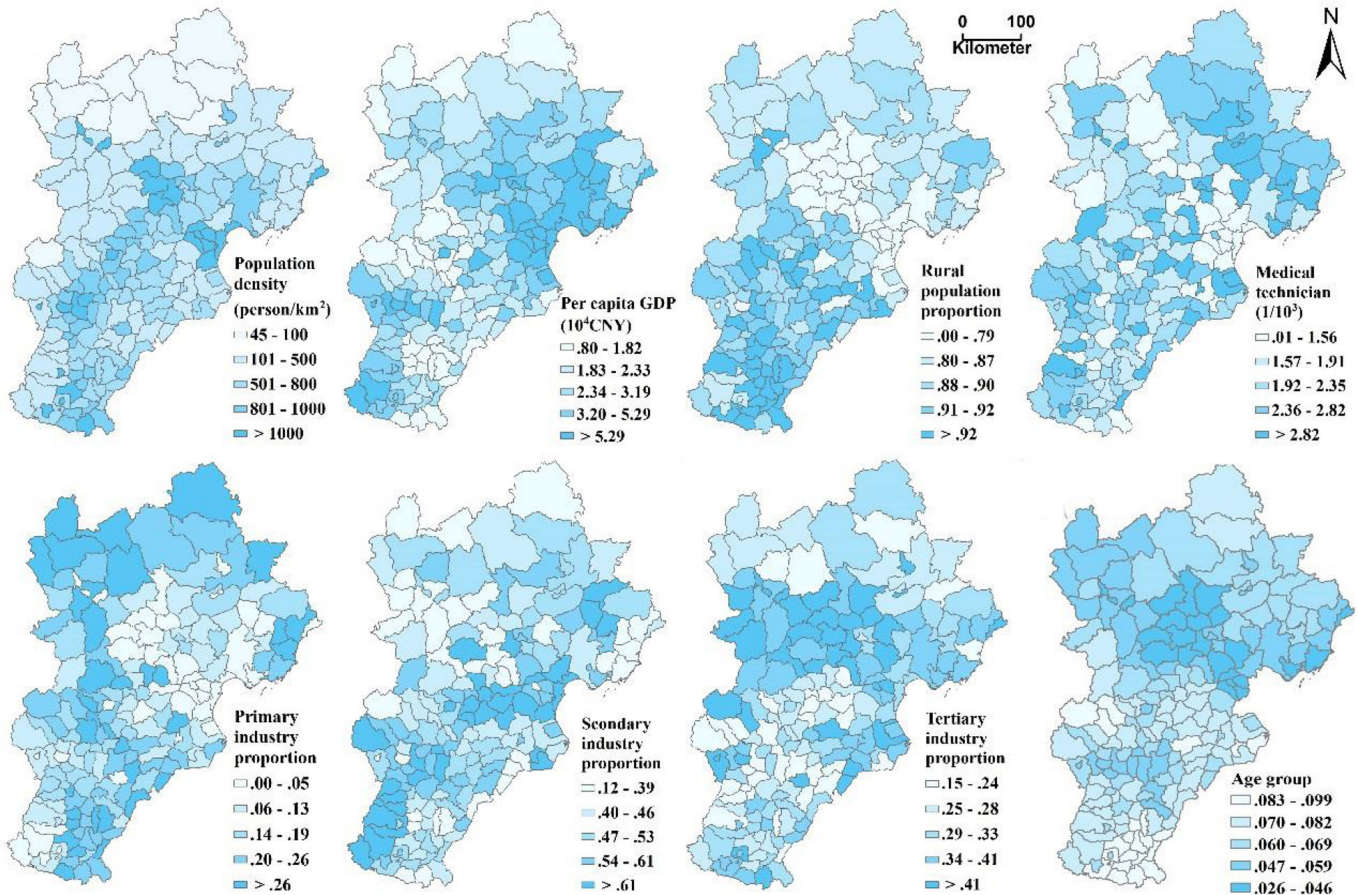
Spatial distribution of bacillary dysentery and socio-economic factors in the counties of Beijing, Tianjin and Hebei

Figure 3 shows the spatial distribution of socio-economic factors in the Beijing–Tianjin–Hebei region. The regions with the highest population density are mainly located in the Beijing and Tianjin urban area. Beijing and its neighbouring areas have a lower proportion of rural population compared with the large area of Hebei, especially in the southern area. The high per capita GDP counties are mainly located at the east of the study area, including Beijing, Tianjin and the coastal area of Hebei. The primary industry is mainly located in the northern mountain area, the secondary industry is mainly located in the mid and southern plain area, and the tertiary industry is mainly located in large urban agglomerations in Beijing and its neighbouring areas. Medical and technical personnel are mainly located in the northeast area of the study region.

The age and gender distribution of bacillary dysentery, as well as socio-economic factors and meteorological factors have been summarised (Table 1). The total number of cases comprised 36,472 in 2012 in the study region, and the average number for each county was 213. Male cases comprised 21,377, accounting for 58.6% of the total number of cases, while female cases comprised 15,095.

**Table 1:** Statistical description of incidence and potential risk factors of bacillary dysentery

Variables	Sum	Mean ± SD	Frequency distribution				
			P(5)	P(25)	P(50)	P(75)	P(95)
Total cases	36,472	213 ± 803	4	21	46	134	710
Male cases	21,377	104 ± 205	1	12	28	79	512
Female cases	15,095	73 ± 157	1	7	17	45	416
0–14 years cases	9999	46 ± 94	0	4	13	41	225
15–59 years cases	21,385	99 ± 230	0	7	18	54	626
≥60 years cases	5088	24 ± 52	0	2	6	17	145
Sunshine (hour)	2462.98	6.73 ± 3.36	0.59	3.90	7.68	9.38	11.21
Mean temperature (°C)	/	10.44 ± 12.14	−8.12	−1.34	13.65	21.30	25.96
Relative humidity (%)	/	57.52 ± 16.61	29.96	43.70	60.12	70.90	81.72
Mean wind (m/s)	/	2.21 ± 0.68	1.31	1.68	2.11	2.60	3.60
precipitation (mm)	/	1.86 ± 5.52	0.00	0.00	0.02	0.99	9.43
Per capital GDP (10^4^CNY)	/	3.96 ± 4.085	1.19	1.96	2.96	4.68	10.15
Population density (person/km2)	/	970.48 ± 2463.954	85.83	354.61	592.39	832.22	2962.62
medical and technical personnel per thousand person	27.26	0.16 ± 0.281	0.03	0.06	0.09	0.15	0.50
Proportion of rural population	/	0.82 ± 0.19	0.31	0.83	0.89	0.92	0.96
Proportion of primary industry	/	0.17 ± 0.108	0.01	0.08	0.17	0.25	0.36
Proportion of secondary industry	/	0.49 ± 0.126	0.27	0.41	0.51	0.58	0.68
Proportion of tertiary industry	/	0.34 ± 0.115	0.20	0.26	0.31	0.39	0.56

The study region has a temperate continental monsoon climate, with an average temperature of 10.44 °C and average precipitation of 1.86 mm. At present, there is strong seasonal variation in a year; for example, the standard deviation of temperature is 12.14 °C, and its 5th, 50th and 95th percentiles are −8.12 °C, 13.65 °C and 25.96 °C, respectively. The other meteorological factors, such as precipitation, wind speed, sunshine hours and relative humidity, also present apparently temporal variations.

In the study, the socio-economic levels present apparent regional heterogeneity; for example, the average proportion of the rural population in the region is 0.82, while its 5th and 95th percentiles are 0.31 and 0.96, respectively. Other potential socio-economic risk factors also have similar characteristics.

In the study, all the variables were linked by the name of each county on a map. The data on the socio-economic characteristics of the study subjects and the disease were aggregated for each county.

### GeoDetector model

The theory of GeoDetector was postulated that if *X* (risk factors) causes *Y* (disease), the associated spatial and temporal distributions tend to be consistent. Distinctive variation in disease incidence is observed depending on the season due to meteorological differences. Meanwhile, there also exhibits spatial heterogeneity of disease incidence level, which resulted from regional difference of impact factors such as socio-economic conditions et al. GeoDetector was used to assess the impact of risk factors on the incidence of bacillary dysentery according to the consistency of the temporal and spatial variations.

The overall influence of each factor and their interactive effect can be qualified by the index of *q* value in the GeoDetector model [21]. It has the advantage of no assumption of linearity and it is not influenced by the collinearity of multivariable.

The input data of GeoDetector model included an explained variable and its stratified information. The parameters preparation and calculation process are as follows. Firstly, the spatio-temporal data were collected. In the study, social variables are stable in time within a year, while the meteorological variables have apparently temporal variation. Then, the strata information were prepared and *q* value was calculated, and the spatial and temporal information of variables can be reflected by stratification [21]. The q value can be expressed as:

$$ q=1-\frac{1}{\mathrm{\Re}{\sigma}^2}{\sum}_{h=1}^L{\mathrm{\Re}}_h{\sigma}_h^2 $$

where *q* denotes the determinant power of a risk factor; the range of the value is between 0 and 1, it means the selected factor explain *q* × 100% of the disease, the bigger the value, the more the determinant power of the factor. If the *q* value is 0, it means that the selected factor is completely unrelated to the disease. On the contrary, if the *q* value is 1, it means that the selected factor is completely related to the disease. $\mathfrak{R}$ is the size of the target spatial-temporal field and *σ* ^2^ denotes the variance of disease incidence in the study area. The stratification was implemented using a discretization process [22]. In the current study, $\mathfrak{R}$ is represented by space and time. *σ* ^2^ is the variance over all the statistical units in the study region and ${\sigma}_h^2$ is the variance within stratum *h*.

The linear contribution of a factor can be measured by *R* ^*2*^ using the linear method chosen, e.g., ordinary least-squares (OLS) regression. However, in a real-world scenario, an assumption of linearity is usually incorrect. As a spatial variance analysis method, the *q* value of GeoDetector is indicative of the non-linear contribution of a risk factor.

In addition to expressing the contribution of single risk factors, the GeoDetector model can also be used to qualify the determinant power of the interaction between two factors. The interactive effect between two selected factors is compared with the effect of their independent contribution to the disease. The classification of the interactive effect is expressed as:
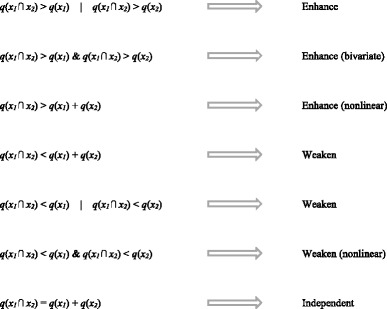


where the symbol of “⋂” means the interaction of two factors, *x*_*1*_ and *x*_*2*_, *q*(*x*_*1*_ ⋂ *x*_*2*_) present the *q* value of the interaction between two factors. *x*_*1*_ ⋂ *x*_*2*_ can be implemented by using the overlay operation for the two layers, *x*_*1*_ and *x*_*2*_, in Geographic Information System (GIS) software, e.g., ArcGIS®. *q*(*x*_*1*_) and *q*(*x*_*2*_) present the *q* value of the independent effect. The symbol of “|” present “or”. The symbol of “&” present “and”. In the calculation of interaction effect *q*(*x*_*1*_ ⋂ *x*_*2*_), the size of target spatial-temporal field ${\mathfrak{R}}_h$ and variance ${\sigma}_h^2$ were estimated in stratum *h* formed by the overlay operation, which have the same meaning as those in the estimation of *q*(*x*_*1*_) or *q*(*x*_*2*_) [21]. The tool was obtained from the GeoDetector website [23]. In the table, the word “nonlinear” means that the interaction between two factors (*q*(*x*_*1*_ ⋂ *x*_*2*_)) is not linear combination of two factors (*q*(*x*_*1*_) and *q*(*x*_*2*_)).

## Results

### Descriptive analysis

**Fig. 4:**
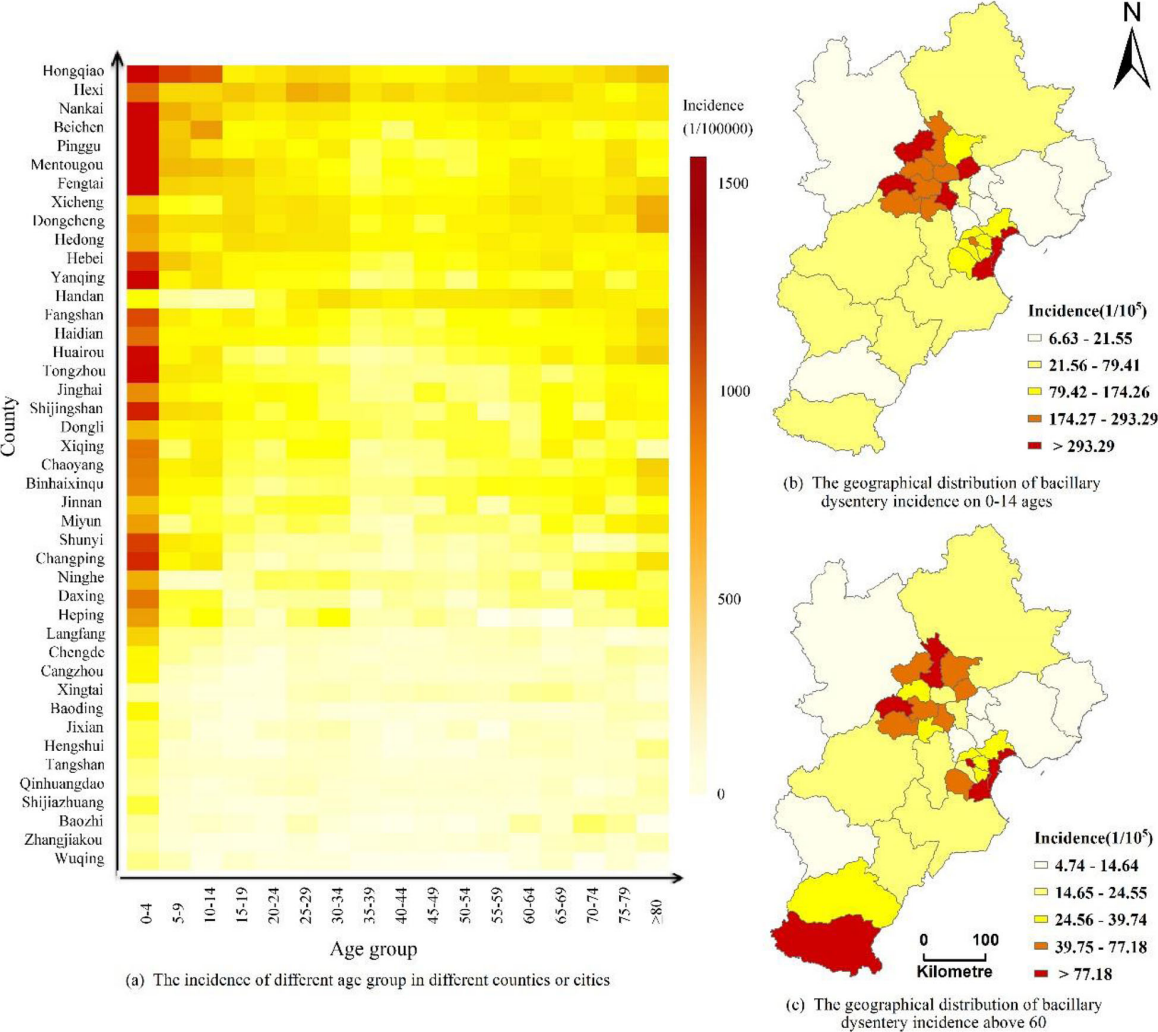
The incidence of bacillary dysentery in different age groups in Beijing, Tianjin and Hebei (left) and spatial distribution of bacillary dysentery incidence for ages 0–14 and over 60 (right). **a** The incidence of different age group in different countries or cities. **b** The geographical distribution of bacillary dysentery incidence on 0-14 ages. **c** The geographical distribution of bacillary dysentery incidence on above 60

The incidence of bacillary dysentery for each typical age group was calculated (Fig. 4). Children are the group at highest risk. For example, the mean incidence of bacillary dysentery in the group under the age of five is 365.12 per 100,000 population, and the highest incidence of the group is 1682.51 per 100,000 population in the Hongqiao county in Tianjin. The average incidence for groups under the age of 14 mainly appeared in the area of Beijing and Tianjin, where the incidence is 229.31 per 100,000 population, while the value is about 29.75 per 100,000 population in the other regions. Older people are the other group at high risk of bacillary dysentery. The spatial regions for high incidence in the group of people aged over 60 also appeared in Beijing, Tianjin and southern Hebei.

### Results from GeoDetector

Individual socio-economic factors and their interactive effects were calculated using the GeoDetector model. General direction of the association between two variables, e.g., a positive or negative relationship, was assessed by Pearson correlation coefficients.

The results show that, the top four factors with the highest determinant power are age group, per capita GDP, population density and proportion of rural population.

Some study has been reveal that bacillary dysentery primarily affects children under the age of 5 years [13, 24]. In the study, the proportion of the population group under age 5 was selected as a factor to assess its contribution to the disease. Results show that the determinant power of the proportion of age group under 5 is strongly associated with transmission of bacillary dysentery, the GeoDetector *q* values is 0.61.

Per capita GDP, population density and proportion of rural population also have apparently association with the disease, and their corresponding q values are 0.27, 0.25 and 0.21, respectively. High per capita GDP was associated with a high incidence of bacillary dysentery; the Pearson’s correlation between them is positive (*r* = 0.17 with the *p* value of 0.03). High population density was also associated with a high incidence of bacillary dysentery; the Pearson’s correlation between them is also positive (*r* = 0.31 with the p value <0.01). A high proportion of rural population proportion was associated with low incidence of bacillary dysentery; the Pearson’s correlation between them is negative (*r* = −0.32 with the *p* value <0.01).

The other selected potential socio-economic risk factors also have a non-negligible effect. The determinant power of the proportion of primary, secondary and tertiary industry is also associated with transmission of bacillary dysentery; their GeoDetector *q* values are 0.09, 0.10 and 0.07, respectively. The determinant power of medical and technical personnel per thousand persons is 0.06.

The results of the GeoDetector interactive effect shows that any two combined factors of age group, per capita GDP, population density and proportion of rural population play a more important role in the transmission of bacillary dysentery. For example, the determinant power of the interaction between age group and other factors are more than 0.6; the determinant power of the interaction between per capita GDP and population density is 0.5; the other two combinations of the interactive effect between per capita GDP and the proportion of the rural population, and population density and the proportion of the rural population are also 0.5. Compared with their independent impact, they all present the effect of “bivariate enhance”. This suggests the need to pay more attention to regions with one or more features of high population density, high per capita GDP and a low proportion of rural population (Table 2).

**Table 2:** The determinant power of single socio-economic factors and their interactive effects on bacillary dysentery (EB: Enhance (bivariate), EN: Enhance (nonlinear))

	Age	GDP per capita	Popu. den.	Rural popu.	Sec. ind.	Prim. ind.	Tert. ind.	Med. staff
Age	0.61							
GDP per capita	0.73^EB^	0.27						
Popu. Den.	0.73^EB^	0.50^EB^	0.25					
Rural popu.	0.71^EB^	0.50^EN^	0.50^EN^	0.21				
Sec. ind.	0.63^EB^	0.39^EN^	0.36^EN^	0.30^EB^	0.10			
Prim. ind.	0.67^EB^	0.36^EB^	0.42^EN^	0.27^EB^	0.22^EN^	0.09		
Tert. ind.	0.65^EB^	0.37^EN^	0.40^EN^	0.23^EB^	0.13^EB^	0.13^EB^	0.07	
Med. staff	0.64^EB^	0.34^EN^	0.32^EN^	0.24^EB^	0.16^EB^	0.18^EN^	0.13^EB^	0.04

Note: Age presents proportion of the population group under age 5 (%), Popu. den. presents population density, Rural popu. presents proportion of the rural population (%), Prim. ind. presents proportion of the primary industry (%), Sec. ind. presents proportion of the secondary industry (%), Tert. ind. presents proportion of the tertiary industry (%), and Med. staff presents medical and technical personnel per thousand persons

**Fig. 5:**
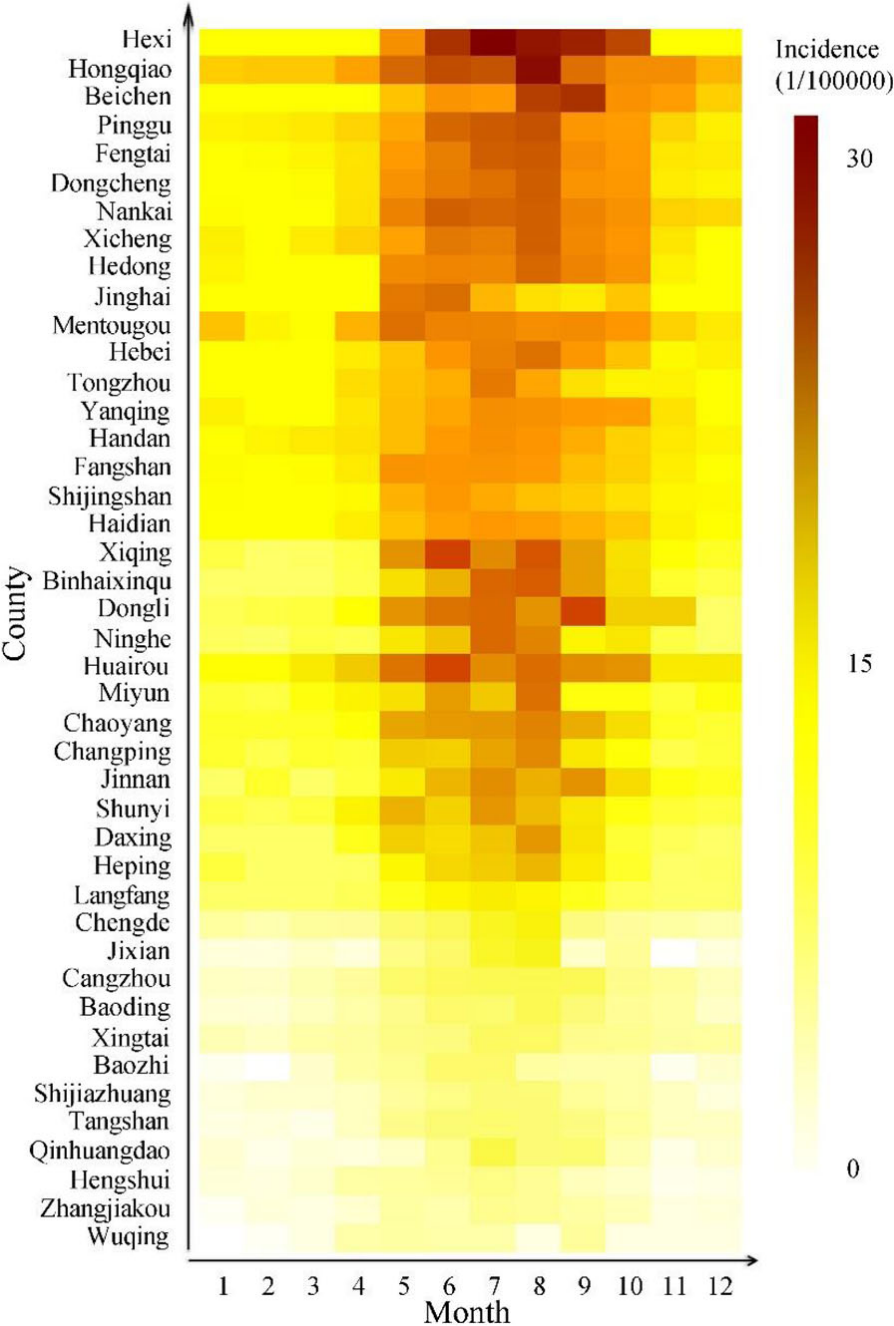
The seasonal variant of bacillary dysentery incidence in each region

Bacillary dysentery incidence presents apparent seasonal variance, with the highest incidence in summer, especially from June to September (Fig. 5). The study finds that some meteorological factors have a higher determinant power on the temporal variant of bacillary dysentery.

The average temperature has the strongest association with bacillary dysentery, where the *q* value of GeoDetector is 0.83. High temperature is associated with high incidence of bacillary dysentery; the Pearson’s correlation between them is positive (*r* = 0.87 with the *p* value <0.01). Mean relative humidity and precipitation have a similar determinant power; their GeoDetector *q* value is 0.31 and 0.25, respectively. High mean relative humidity and precipitation are related to high incidence of bacillary dysentery; the Pearson’s correlation between bacillary dysentery incidences are all positive, too (*r* = 0.50 with *p* < 0.01 and *r* = 0.29 with p < 0.01), respectively.

The variables of wind speed and sunshine hours have a relative lower effect on bacillary dysentery, and the GeoDetector *q* values are 0.17 and 0.13, respectively. High wind speed is associated with low bacillary dysentery incidence (the Pearson’s correlation is negative *r* = −0.23), while high sunshine hours are associated with high bacillary dysentery incidence (Pearson’s correlation is positive *r* = 0.06).

The results of the GeoDetector interactive effect show that the combined impact of temperature and other potential risk factors play an important role in the transmission of bacillary dysentery. For example, the determinant power of the interaction between temperature and relative humidity is 0.87, the determinant power of the interaction between temperature and wind speed is 0.86, the determinant power of the interaction between temperature and precipitation is 0.84. They all show the “bivariate enhance” effect compared with their independent influence. This suggests the need to pay more attention to periods with high temperature and relative humidity, or conditions with lower temperature and high wind speed (Table 3).

**Table 3:** The determinant power of single meteorological factors and their interactive effect on bacillary dysentery (EB: Enhance (bivariate), EN: Enhance (nonlinear))

	Temperature	Relative humidity	Precipitation	Wind speed	Sunshine hours
Temperature	0.83				
Relative humidity	0.87^EB^	0.31			
Precipitation	0.84^EB^	0.40^EB^	0.25		
Wind speed	0.86^EB^	0.44^EB^	0.44^EN^	0.17	
Sunshine hours	0.84^EB^	0.56^EN^	0.42^EN^	0.31^EN^	0.13

## Discussion

The Beijing–Tianjin–Hebei region is the largest urban agglomeration in north China, which is centred on the capital, Beijing. In 2012, there were 36,472 cases of bacillary dysentery in the region, which is apparently more than in other areas of China. The epidemiological characteristics and socio-economic and meteorological risk factors have been analysed in the study. The results show that children are the high-risk group for the disease. Transmission of bacillary dysentery is influenced by both the physical and the socio-economic environment [12, 13].The disease incidence has apparent high spatial heterogeneity due to the influence of socio-economic factors, in which per capita GDP, population density and the proportion of the rural population are strongly associated with the transmission of bacillary dysentery. Meanwhile, the disease incidence has an apparent high temporal variant due to the influence of meteorological factors in which the temperature, relative humidity and precipitation have a significant influence.

A previous study on spatial-temporal patterns and a risk factor analysis of bacillary dysentery was carried out in the Beijing-Tianjin-Tangshan urban region of China [13]. The setting for the previous study was only a fifth the area covered by the current study, and the former focused on the most developed urban area only. In the current study, a wide rural area around the urban region was also included. In the previous study, the area at risk of bacillary dysentery was identified using SaTScan method. The linear effects in relation to the risk factors were analyzed accordingly. By comparison, the area at risk of this disease in the current study was identified using GeoDetector method, and the nonlinear effects pertaining to the risk factors were evaluated.

In the study, epidemiological characteristics analysis demonstrated that the risk in children was much higher compared with other age groups, and the peak period of incidence is in summer and autumn, especially in the months around August. These results are consistent with other studies [25, 26]. The summer holiday period may pose a risk, in the period children leave kindergarten or schools with good sanitation, and some may travel with parents to places with high population density, which presents more opportunities for exposure to bacteria.

Bacillary dysentery incidence presents an apparent seasonal variance; climatic variation is only one of several important factors influencing the transmission of shigella. The study shows that temperature has the highest determinant power of the disease, which is 0.83, and high temperature is associated with high incidence. This is consistent with previous studies. Higher temperature periods are conducive for the replication and spread of bacteria due to people eating more rapidly deteriorating food [9]. For example, in the northern city, Jinan, there is an 11% increase in bacillary dysentery incidence if the temperature increases by 1 °C [8]. The situation is similar to that of Lima in Peru, where it has been found that risk increased by 8% with an increase of 1 °C temperature [24].

In addition to temperature, relative humidity and precipitation are another two important climate factors influencing the transmission of bacillary dysentery. The study shows that high relative humidity and precipitation are associated with high incidence; their determinant powers with regard to the disease are 0.31 and 0.25, respectively. Air relative humidity is a key factor influencing food preservation; for example, in dry desert areas, food can last longer and there are fewer flies and other media to transmit infection. Precipitation also helps to spread bacillary dysentery through people drinking polluted water, especially in conditions of poor sanitation and crowded living. Previous studies also have similar findings in other regions. For example, Huang et al. found that there was a positive association between precipitation and the spread of bacillary dysentery [7].

The study region is located in a temperate continental monsoon climate zone. High temperature is accompanied by high precipitation and high air relative humidity. These meteorological factors present a significant interactive effect. The determinant power of the combination of temperature and relative humidity is 0.87, which implies that a humid and hot environment will exacerbate transmission of the disease by affecting the replacement speed of pathogens. The determinant power of the combination of temperature and wind speed is 0.86.It was demonstrated in the current study that strong winds and low temperatures inhibited the spread of bacillary dysentery, while disease transmission was expedited by high temperatures and gentle winds. A possible interpretation of this is that low temperatures inhibit the reproduction of bacteria, while strong winds facilitate evaporation, this enables dry food to be kept, result in reduction of bacterial growth. In addition, the opportunity for frequency of contact between infected people and others is reduced in environmental conditions where strong winds and low temperatures feature, thus also inhibiting disease transmission.

Compared with meteorological factors, socio-economic development levels present significant spatial heterogeneity, and they have been analysed as the key potential factors responsible for the spatial variant of bacillary dysentery incidence. Age group has strong association with the disease, the implication of this is that greater attention should be paid to vulnerable groups identified as being at high risk of bacillary dysentery. The other factors are proxy variables for the environment of bacterial breeding, transmission, individual hygiene conditions and public health medical facilities. They are all positively associated with disease incidence. Per capita GDP, as a proxy for individual hygiene conditions, has the highest determinant power amongst these socio-economic factors. The other factors of population density and proportion of rural population represent living conditions, and they also play an important role.

The interactive effect between per capita GDP and the other factors of population density and the proportion of the rural population were enhanced compared with the effect from their independent effects. In the study regions, the area features high per capita GDP, more population density and a lower proportion of rural people mainly located in the urban area of the city. Accordingly, there is less living and work room, which means more opportunity for contact between people. Furthermore, huge influxes of people, especially migrant workers in the urban area, live in crowed rooms with poor sanitary conditions, which also facilitate spread of the disease. This implies that a strategy for bacillary dysentery control should focus on improving sanitation conditions and should also focus on locations with high population densities.

In the study, GeoDetector method was used to assess the impact of risk factors associated with bacillary dysentery on inhabitants in the selected region and to determine high-risk regions and seasons. The use of GeoDetector method has numerous advantages compared with the traditional methods used, e.g., OLS regression or spatial regression models. Firstly, an assumption is not made about the probability distribution of the data used in the calculation, in contrast to the regression model which includes 10 exacting assumptions, one of which is that a probability distribution of the dependent variables, e.g., normal distribution, is required. Secondly, with the use of GeoDetector, any association between the explanatory variables (x) and the dependent variables (y) is real; there is no assumption of linear or nonlinearity. Thirdly, the explanatory variables (x) can be either categorical or numerical with the method we selected, whereas numerous categorical variables pose a challenge when using the regression model [27]. Fourthly, the q value in GeoDetector is intuitive, can easily be explained and has different perspective in contrast to how it is used in OLS regression. For example, with GeoDetector, an explanatory variable is able to explain *q* × 100% of the dependent variables. By contrast, the regression coefficient represents the rate of change of the dependent variables in relation to the explanatory variables with the use of the regression model.

In practical application, as is the case with the use of other traditional methods, the use of GeoDectector involves certain challenges. For example, if two variables, e.g., population density and urbanity, are applied to address a similar mechanism, the explanation of the results will be affected. Fortunately, the GeoDetector findings were not affected by collinearity between multiple explanatory covariates, and the study objective was to identify regions at high risk of bacillary dysentery and to determine the allocation of medical resources to the public in this regard. Thus, although two variables were applied to address the same mechanism, they could still be attributed to the same region or identified within a certain period.

There were also some uncertainties in the study, the potential risk factors were collected on a large scale; for example, the economic factors were aggregated at the county level. However, transmission of the disease is more influenced by personal hygiene habits and community health conditions [20]. Future work will entail collecting more detailed information about people’s environments (e.g. their indoor living space) and their community environment (e.g. public green space and the spatial distribution of refuse bins). In addition, the method used in the study has limitation in capturing partial correlations between dependent variable and independent variables, this would introduce uncertainties in the results. Furthermore, in the epidemiological studies, the direct variables effecting the disease transmission usually can not be measured, so that in the study, the corresponding proxy variables were used, this would also introduce some uncertainty.

## Conclusions

The study reveals that in Beijing–Tianjin–Hebei, bacillary dysentery is mainly apparent in the population of children. Transmission of the disease is affected by meteorological and socio economic factors. In particular, it is necessary to focus on the hot and humid period and places in urban areas with high population density and a low proportion of the rural population.
